# The Effects of *Limosilactobacillus reuteri* LR-99 Supplementation on Body Mass Index, Social Communication, Fine Motor Function, and Gut Microbiome Composition in Individuals with Prader–Willi Syndrome: a Randomized Double-Blinded Placebo-Controlled Trial

**DOI:** 10.1007/s12602-021-09800-9

**Published:** 2021-06-11

**Authors:** Xue-Jun Kong, Kevin Liu, Patrick Zhuang, Ruiyi Tian, Siyu Liu, Cullen Clairmont, Xiaojing Lin, Hannah Sherman, Junli Zhu, Yelan Wang, Michelle Fong, Alice Li, Bryan K. Wang, Jinghan Wang, Zhehao Yu, Chen Shen, Xianghua Cui, Hanyu Cao, Ting Du, Guobin Wan, Xia Cao

**Affiliations:** 1 Athinoula A. Martinos Center for Biomedical Imaging, Massachusetts General Hospital, Boston, MA USA; 2 Department of Medicine and Psychiatry, Beth Israel Deaconess Medical Center, Boston, MA USA; 3 PWS Care and Support Center, Hangzhou, China; 4 Yale University, New Haven, CT USA; 5 Brandeis University, Waltham, MA USA; 6 New York University, New York, NY USA; 7 The Second Affiliated Hospital of Kunming Medical University, Kunming, Yunnan China; 8 Shenzhen Maternity and Child Healthcare Hospital, Shenzhen, Guangdong China

**Keywords:** Prader–Willi syndrome, Body mass index (BMI), Social communication, Fine motor function, Microbiome, *Limosilactobacillus reuteri* (*Lactobacillus reuteri*)

## Abstract

Prader–Willi syndrome (PWS) is a rare genetic disorder associated with developmental delay, obesity, and neuropsychiatric comorbidities. *Limosilactobacillus reuteri* (*Lactobacillus reuteri*, *Lact. reuteri*) has demonstrated anti-obesity and anti-inflammatory effects in previous studies. In the present study, we aim to evaluate the effects of *Lact. reuteri* supplementation on body mass index (BMI), social behaviors, and gut microbiota in individuals with PWS. We conducted a 12-week, randomized, double-blind, placebo-controlled trial in 71 individuals with PWS aged 6 to 264 months (64.4 ± 51.0 months). Participants were randomly assigned to either receive daily *Lact. reuteri* LR-99 probiotic (6 × 10^10^ colony forming units) or a placebo sachet. Groupwise differences were assessed for BMI, ASQ-3, and GARS-3 at baseline, 6 weeks, and 12 weeks into treatment. Gut microbiome data was analyzed with the QIIME2 software package, and predictive functional profiling was conducted with PICRUSt-2. We found a significant reduction in BMI for the probiotic group at both 6 weeks and 12 weeks relative to the baseline (*P* < 0.05). Furthermore, we observed a significant improvement in social communication and interaction, fine motor function, and total ASQ-3 score in the probiotics group compared to the placebo group (*P* < 0.05). Altered gut microbiota was observed in the probiotic group to favor weight loss and improve gut health. The findings suggest a novel therapeutic potential for *Lact. reuteri* LR-99 probiotic to modulate BMI, social behaviors, and gut microbiota in Prader–Willi syndrome patients, although further investigation is warranted.

*Trial registration* Chinese Clinical Trial Registry: ChiCTR1900022646

**Supplementary Information:**

The online version contains supplementary material available at 10.1007/s12602-021-09800-9.

## Introduction

Prader–Willi syndrome (PWS) is an uncommon genetic syndrome that affects approximately one out of every 15,000 people [1]. PWS is recognized as the most common genetic cause of life-threatening childhood obesity and often presents with neuropsychiatric symptoms, such as emotional lability, compulsive behavior, and psychosis [1, 2]. Morbid obesity and neuropsychiatric complications are leading causes of death or long-term disabilities. Besides some reported efficacy of growth hormone [3], the treatments for PWS are mainly behavioral.

The gut microbiota has been implicated in the pathogenesis of obesity and the neuropsychiatric comorbidities in PWS subjects [4]. Although the gut dysbiosis was found to be quite similar in PWS-related obesity and diet-related obesity [5], a recent study identified several bacteria genera that are differentially abundant in the PWS population [6]. Interestingly, when dysbiotic gut microbiota from PWS patients were transplanted into mice, insulin-receptor signaling decreased 2 weeks prior to an increase in body fat composition, indicating that the gut microbiome in PWS may play a role in the development of obesity [7]. Dysbiosis of the gut microbiome has also been associated with neuropsychiatric conditions, such as psychotic and affective disorders [8]. Past research conducted in our laboratory indicated that the microbiome composition even has the potential to serve as a biomarker in the diagnosis and subtyping of ASD [9].

Recent research has shown the potential for probiotics to improve the most debilitating symptomology of PWS. Gut microbiome composition and metabolic disturbance were improved after probiotic administration in overweight adults [10]. However, the effect of probiotics on obesity and in the PWS population remains under debate. An investigation of the probiotic *Bifidobacterium lactis* in PWS revealed no significant changes in stool frequency or microbiome composition [11]. While studies have found positive effects of *Limosilactobacillus reuteri* (*Lactobacillus reuteri*, *Lact. reuteri*), the probiotic strain administered in this study, on inflammatory and metabolic diseases [12, 13]; the effect of *Lact. reuteri* is also uncertain, as one study did find a positive correlation between the endogenous abundance of *Lact. reuteri* and body mass index (BMI) in Mexican children [14]. Notably, a recent study found that *Lact. reuteri* administration in the PWS population improved fasting insulin concentration and insulin sensitivity and decreased abdominal adiposity in children older than 4.5 years of age [15]. *Lactobacillus reuteri* has also been shown to exert beneficial effects on the brain and behavior [16]. Previous studies have reported that *Lact. reuteri* upregulates the neuropeptide hormone oxytocin (OXT), a factor integral to social bonding and reproduction, within a vagus nerve-mediated pathway in mice [17, 18] and humans [19]. Additional research has found that *Lact. reuteri* acts in a vagus nerve-dependent manner to rescue deficits in social interaction-induced synaptic plasticity via oxytocin signaling modulation in multiple animal models of ASD [20]. Preliminary results reported from the phase III CARE-PWS clinical trial indicated beneficial effects of intranasal oxytocin treatment on hunger, anxiety, and obsessive-compulsive behaviors in patients with PWS; however, this finding has not yet been published. *Lactobacillus reuteri* probiotic treatment has potential to be another therapeutic option for upregulating oxytocin in patients with PWS and thereby similarly improving symptomology.

In this study, we conducted a randomized, double-blind, placebo-controlled trial to test our hypothesis that probiotics consumption has beneficial effects on obesity, social behaviors, and neurodevelopment in PWS. We enrolled a total of 71 PWS patients to evaluate the efficacy of a *Lact. reuteri* strain *LR-99* on their BMI, psychological measurements, and gut microbiome compositions and functions relative to placebo controls. In addition to potentially supporting a new intervention for patients with PWS, the microbiome composition data collected from this study may shed light on the underlying mechanisms of PWS pathology and the gut–brain axis.

## Materials and Methods

### Study Design

**Fig. 1:**
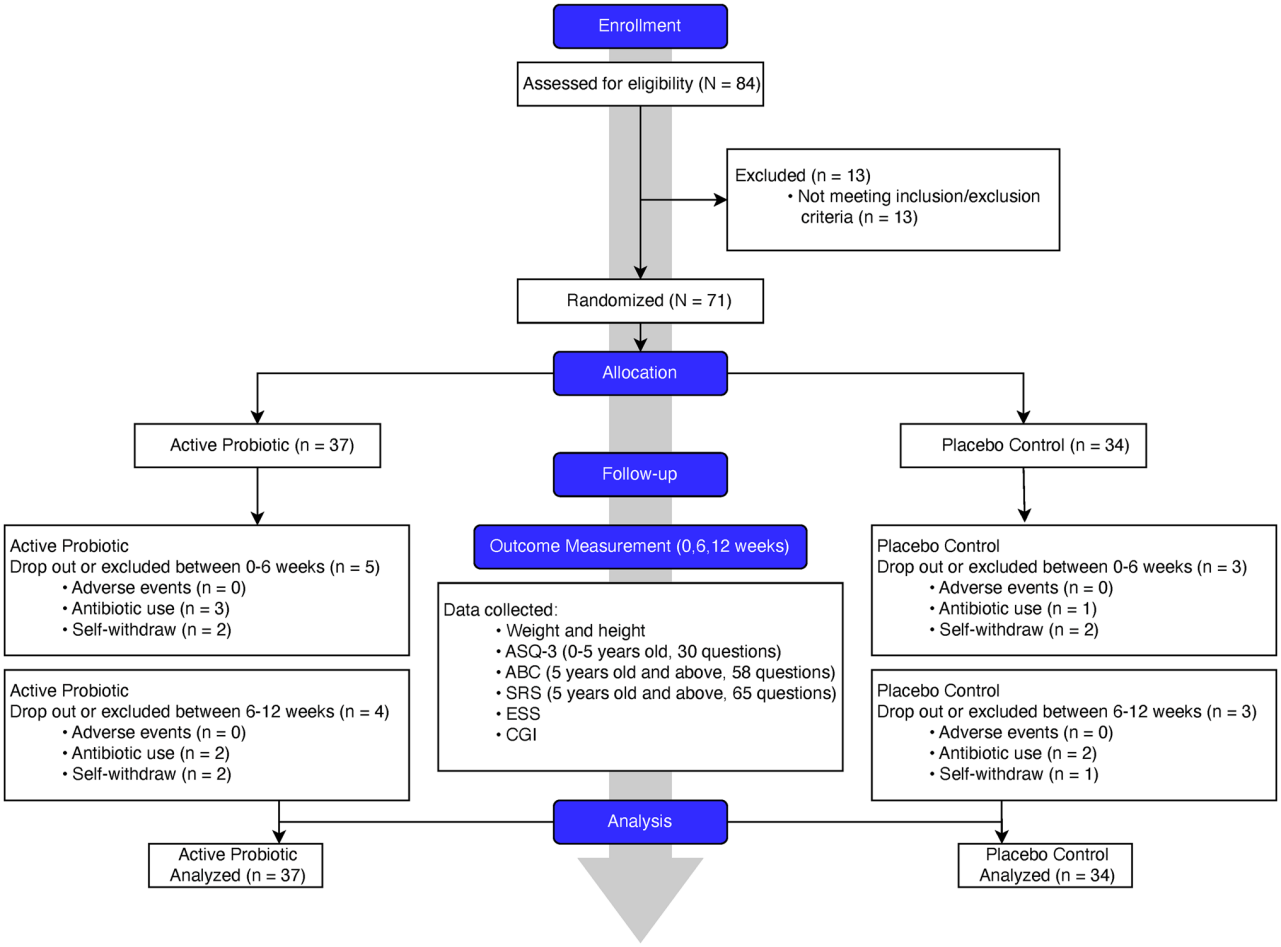
Flowchart summary of study conduct and participant enrollment and dropout

We designed and conducted a randomized, double-blinded, placebo-controlled clinical trial (flowchart, Fig. 1). In this trial, we randomly assigned enrolled PWS participants, with a 1:1 ratio, to either the probiotics or placebo group. We hypothesize that a 12-week treatment period is sufficient for probiotics supplementation to induce detectable changes. To achieve a statistical power of 80% for primary outcomes with a large effect size of 0.8 (Cohen’s *d*) assumed, a total of 52 participants (26 in each arm) were required. We enrolled and randomized 71 subjects (probiotics group = 37, placebo group = 34), and all subjects with collected data were included in the final intention-treat data analysis.

### Ethical Considerations

The study was conducted in accordance with the Declaration of Helsinki. Ethical approval was issued by the Internal Review Board (IRB) of the Second Affiliated Hospital of Kunming Medical University, Kunming, China (Review-YJ-2016-06). The present clinical trial of probiotics was registered at the Chinese Clinical Trial Registry (ChiCTR) with registration number ChiCTR1900022646, in which two interventions of different probiotic strains were independently evaluated in the enrolled subjects. The evaluation of effects of the alternative probiotic strain *Bifidobacterium Animalis* subsp. *lactis BB-11*, which was co-registered with the present study, was previously published [21]. Signed informed consent was obtained from the parents or legal guardians of all the subjects according to the IRB requirements.

### Participants

Study participants were recruited through the PWS Care & Support Centre, located in Zhejiang, China. Participants were included if they met the following criteria: they had been genetically confirmed to have PWS, had not been administered any forms of probiotics for at least 4 weeks, had stable medications for at least 4 weeks, had no planned changes in medications or psychosocial interventions during the trial, had a willingness to provide stool samples in a timely manner, and had a willingness to cooperate with interviews and study procedures. Potential participants were excluded if they had other known genetic disorders, or if they were pregnant or breast-feeding before the study.

### Randomization and Blinding

Randomization and allocation concealment were performed by a statistician who was not part of the research team. Randomization sampling numbers were electronically generated for each deidentified subject. Coded probiotics and placebo of identical appearance were prepared by Beijing Huayuan Academy of Biotechnology to ensure allocation concealment. Both the participants and the research staff/investigators who collected and analyzed the outcome data were blinded to treatment status. Blinding was also maintained by making the probiotics package appear identical to the placebo sachet.

### Intervention

The probiotic *Limosilactobacillus reuteri* LR-99 (Beijing Huayuan Academy of Biotechnology) was used in the study in the format of a sachet. Each sachet of probiotics supplement contained 3 × 10^10^ colony forming units (CFUs). The placebo was maltodextrin in the sachet with similar color, flavor, and taste as the probiotic sachets. Subjects received one sachet twice a day of either probiotics or placebo for a duration of 12 weeks and were instructed to consume the sachet contents orally with water.

### Outcomes

#### Primary Outcomes

Weight and height measurements were obtained by parents using household scales and collected by the research staff. BMI calculated by weight and height was converted to z-score using age growth references provided by WHO (2006) [22].

Psychological questionnaires were administered by trained and qualified study staff that are bilingual in both English and Mandarin Chinese. The administration of such questionnaires was conducted via interview in Mandarin Chinese.

- Ages and Stages Questionnaires, 3rd Edition (ASQ-3) [23]
  The ASQ-3 is one of the most widely available development screening tools for young children. The ASQ-3 has five domains: communication, gross motor, fine motor, problem-solving, and personal–social. Total scores were calculated. We interviewed all subjects younger than 5 years old.
- Gilliam Autism Rating Scale Third Edition (GARS-3) [24]
  The GARS-3 consists of 56 items describing characteristic behaviors of individuals with autism. The items are grouped into six sub-scales: restrictive, repetitive behaviors (RRB), social interaction (SI), social communication (SC), emotional responses (ER), cognitive style (CS), and maladaptive speech (MS). Total scores and sub-scales were calculated. We interviewed all subjects older than 3 years old.

#### Secondary Outcomes: Fecal Microbiome

##### Sample Handling and Collection

Stool samples were collected with DNA/RNA shield fecal collection tubes (Zymo, Cat#R1101) containing 1 mL preservation solution and were transported to the laboratory by ice bags and then frozen at −80 °C. TIANmap stool DNA kit was used to extract DNA (TIANGEN, Cat#DP328) according to the manufacturer’s instructions, and DNA samples were carefully quantified with a NanoDrop Spectrophotometer. A_260_/A_280_ ratios were also measured to confirm high-purity DNA yield. DNA samples were frozen at −20 °C until use.

##### 16S rRNA Gene Amplicon Sequencing

The 16S rRNA V3–V4 library was constructed by two rounds of PCR with the following primers: 341F:5′TCGTCGGCAGCGTCAGATGTGTATAAGAGACAGCCTACGGGAGGCAGCAGCCTACGGGNBGCASCAG3′ and 805R:5′GTCTCGTGGGCTCGGAGATGTGTATAAGAGACAGTGACTACNVGGGTATCTAATCC3′ via reaction procedure (95 °C for 2 min, followed by 25 cycles at 95 °C for 30 s, 55 °C for 30 s, and 72 °C for 30 s, and a final extension at 72 °C for 5 min). PCR products were purified with 1 × KAPA AMPure beads (KAPA, Cat#KK8002). Then, products were put through a second PCR reaction procedure (95 °C for 2 min, followed by 8 cycles at 95 °C for 30 s, 55 °C for 30 s, and 72 °C for 30 s, and a final extension at 72 °C for 5 min). PCR products were purified with 1 × KAPA AMPure beads and analyzed using a Bioanalyzer DNA Kit, followed by quantification with real-time PCR. DNA libraries were pooled and sequenced on Illumina MiSeq (Illumina; CA) using a 2 × 250 bp paired-end protocol with overlapping reads.

### Statistical Analysis

All raw data were recorded and processed in Microsoft Excel 2007 and R. The presentation of data follows the CONSORT recommendations for reporting results of randomized clinical trials (RCTs). Statistical procedures were carried out using α = 0.05 as the significance level, and missing data was omitted during statistical analysis.

Baseline demographic features were compared between groups using Pearson’s χ^2^-test. We applied the Wilcoxon rank-sum test to explore the intergroup differences in the z-scores of weight, height, total scores, and sub-scores of ASQ-3, GARS-3, ABC, and SRS at baseline, per-subject changes from 0 to 6 weeks, and per-subject changes from 6 to 12 weeks.

Primary outcomes were analyzed using linear mixed effect models (LME) to assess for differences within each primary outcome over the course of the study (0–6 weeks, 6–12 weeks, and 0–12 weeks) for each group via the *LMER* package. For all LME analyses, we included time, age, and gender as fixed effects and a random intercept to account for within-subject correlation due to repeated measures over time. In the case of a significant main effect, Bonferroni-corrected pairwise comparisons were conducted.

Secondary outcomes were analyzed using similar methods as that of primary outcomes. In addition, linear regression was performed to check for correlations between clinical indices and microbiome compositions.

Receiver operated characteristic (ROC) curves were constructed via the *plotROC* package for multiple logistic regression models using either select clinical or predictive functional profiling indices.

### Microbiome Data Processing and Analysis

The sequencing reads were filtered using the *QIIME2* (v2019.10) based on quality scores [25]. *Deblur* was used to denoise with default parameters and obtain an abundance table of samples by amplicon sequence variants (ASVs) [26].

Alpha diversity metrics were calculated with *QIIME2*. Bray–Curtis distance was used to characterize microbiome beta diversity. Taxonomies for ASVs were assigned using the *sklearn*-based taxonomy classifier trained on the sequences at 99% similarity level from *Greengenes* v13.8. Significant differences in the relative abundance of microbial phyla, genera, and alpha diversity between placebo and probiotics groups were identified by Kruskal–Wallis tests. A false discovery rate (FDR) based on the Benjamini–Hochberg (BH) adjustment was applied for multiple comparisons.

*PICSRUSt-2* was used to infer microbial functional content based on ASVs’ abundant tables and then produced Kyoto Encyclopedia of Genes and Genomes (KEGG) orthologs (KO), enzyme classification numbers, and pathway abundance table [27, 28]. The differential analyses were performed on the fold ratios between probiotics and placebo group with a permutation-based nonparametric test, and the top differential features were rendered and plotted with *Calour* [29]. *MaAsLin2* was used to explore per-feature correlations between clinical indices and microbial taxa abundances, and results were adjusted for multiple testing using FDR based on the Benjamini–Hochberg method [30].

All raw data from 16 s rRNA Illumina amplicon sequencing have been deposited in The National Centre for Biotechnology Information (NCBI) Sequence Read Archive (SRA, PRJNA643297).

## Results

### Demographic Features of PWS Participants

A total of 71 subjects aged 64.4 ± 51.0 months (ranging from 6 to 264 months) with genetically confirmed diagnosis of Prader–Willi syndrome. Of which, 37 subjects aged 65.0 ± 53.8 months were randomized to receive active probiotic, *Lact. reuteri*, while 34 subjects aged 64.0 ± 49.0 months were randomized to receive placebo. An overview of the subject age distribution is shown in the figure presented in Online Resource 1. Additionally, due to difficulties in data collection, a total of 56 subjects (*n* = 28 in each arm) have available baseline clinical indices. Groupwise comparisons of baseline age, sex, genotype, and other characteristics did not indicate any significant differences (*P* > 0.05). Detailed demographic characteristics of the enrolled participants are summarized in Table 1.

**Table 1:** Demographic features and baseline characteristics of study participants

		Active probiotic (*N* = 37)	Placebo control (*N *= 34)	*P*-value
Age (months, *n* (mean ± SD))	All Subjects	28 (65 ± 53.8)	28 (64 ± 49.0)	1.00
	> 5 years	12 (113 ± 50.2)	11 (113 ± 42.7)	0.81
	≤ 5 years	16 (29 ± 9.8)	17 (32 ± 11.5)	0.52
Sex (*n* (%))	Male	12 (43%)	18 (64%)	0.18
	Female	16 (57%)	10 (36%)	
Genotype (*n* (%))	Deletion	16 (57%)	15 (54%)	0.56
	Disomy	4 (14%)	2 (7%)	
	Other/unknown	8 (29%)	11 (39%)	
Weight (kg, mean ± SD)		25.8 ± 15.3	26.2 ± 21.0	0.71
Height (cm, mean ± SD)		109.6 ± 23.9	107.2 ± 26.6	0.66
BMI (mean ± SD)		19.3 ± 4.58	19.7 ± 6.87	0.53

The study recruitment procedure and dropouts at each study timepoint are illustrated as a flowchart in Fig. 1. No serious or severe adverse events were observed. There were no significant differences found between the two groups in any observed adverse events (*P* > 0.05). Of 71 initially enrolled subjects, 15 dropped over the course of the trial. Eight subjects were dropped due to necessary antibiotics use for mild infection occurrence that is not related to the probiotic, which led to termination; seven of those dropped were due to parent- or self-withdrawal; and none of those dropped were due to adverse effects (Fig. 1).

### Effects of Probiotics on BMI and Psychological Measurements

To assess for within-group longitudinal changes in primary outcomes, we applied Bonferroni-corrected pairwise comparisons on linear mixed effects models, using age, gender, and study timepoint as fixed effects and the subject as a random intercept to account for repeated measures over time. The estimated marginal means of BMI at each study visit are detailed in a table (Online Resource 2). Based on such an analysis, we determined that subjects receiving active probiotic display a significant reduction in BMI. Specifically, such significant differences are uniquely observed in the active probiotic group for BMI between baseline and week 6, and between baseline and week 12 (Table 2, *P* < 0.05).

**Table 2:** Pairwise comparisons of change in BMI at 6 weeks and 12 weeks compared to baseline based on linear mixed effects models

Intervention	Contrast	Mean difference	Standard error	Degrees of freedom	t-Ratio	*P-*Value
Active probiotic	12–6 w	−0.312	0.362	23.153	−0.863	1.000
	12–0 w	−1.291	0.366	30.012	−3.532	0.004
	6–0 w	−0.979	0.361	23.562	−2.710	0.037
Placebo control	12–6 w	−3.912	1.929	17.072	−2.028	0.175
	12–0 w	−1.459	1.782	20.078	−0.819	1.000
	6–0 w	2.453	1.734	18.679	1.414	0.521

Groupwise comparisons of psychological assessment scores were performed at weeks 0, 6, and 12 via the Wilcoxon rank-sum test. A summary of psychological assessment scores, including GARS-3 and ASQ-3, as well as associated statistics for both groups at weeks 0, 6, and 12 are provided in Table 3.

**Table 3:** Summary of psychological measurements, including the ASQ-3 and GARS-3 measures at study timepoints 0-, 6-, and 12-weeks

	0 weeks			6 weeks			12 weeks		
	Placebo control	Active probiotic	*P*-Value	Placebo control	Active probiotic	*P*-Value	Placebo control	Active probiotic	*P*-Value
GARS-3									
Overall severity	2.07 ± 0.46	1.94 ± 0.68	0.550	2.09 ± 0.54	2.08 ± 0.86	0.720	2.00 ± 0.47	1.92 ± 0.76	1.000
Cognitive style (CS)	10.4 ± 4.21	9.63 ± 4.35	0.762	9.36 ± 5.54	10.3 ± 4.17	0.647	8.55 ± 4.11	11.1 ± 5.59	0.120
Emotional responses (ER)	13.5 ± 4.89	13.4 ± 5.19	0.895	14.0 ± 3.46	14.4 ± 6.14	0.703	14.9 ± 2.91	14.3 ± 4.50	0.849
Maladaptive Speech (MS)	5.90 ± 3.42	6.63 ± 5.62	0.955	6.00 ± 3.97	7.85 ± 5.47	0.426	5.09 ± 4.13	6.15 ± 4.86	0.819
Restrictive/repetitive behaviors (RRB)	19.4 ± 7.31	15.9 ± 8.55	0.193	17.8 ± 8.28	20.2 ± 7.63	0.623	18.1 ± 7.52	18.5 ± 9.81	0.820
Social communication (SC)	16.6 ± 6.37	11.7 ± 7.40	0.047	17.8 ± 5.64	12.7 ± 4.96	0.088	18.8 ± 5.93	11.5 ± 6.01	0.007
Social interaction (SI)	11.9 ± 9.20	6.50 ± 4.52	0.082	14.9 ± 10.4	10.1 ± 10.0	0.095	13.1 ± 8.55	7.69 ± 5.57	0.037
ASQ-3									
Total score	119 ± 61.5	131 ± 66.0	0.593	153 ± 53.4	178 ± 44.6	0.485	123 ± 33.3	193 ± 33.8	0.032
Communication	30.5 ± 12.6	33.8 ± 17.1	0.530	42.5 ± 10.8	41.3 ± 10.6	0.415	32.0 ± 15.2	45.0 ± 14.1	0.346
Fine motor	22.5 ± 17.7	20.6 ± 9.43	0.892	22.5 ± 15.4	24.4 ± 17.4	0.686	11.0 ± 8.22	25.0 ± 15.3	0.027
Gross motor	15.0 ± 16.0	26.3 ± 24.2	0.392	28.3 ± 18.6	35.6 ± 14.7	1.000	24.0 ± 10.8	39.3 ± 15.1	0.245
Personal–social	28.0 ± 12.5	26.3 ± 17.9	0.858	29.2 ± 11.6	39.4 ± 13.2	0.197	31.0 ± 12.4	40.7 ± 15.1	0.234
Problem solving	23.0 ± 12.7	23.8 ± 17.5	0.964	30.8 ± 15.3	36.9 ± 14.1	1.000	25.0 ± 12.7	42.9 ± 8.09	0.051

### Changes in Microbiome Composition and Function with Probiotics Intervention

**Fig. 2:**
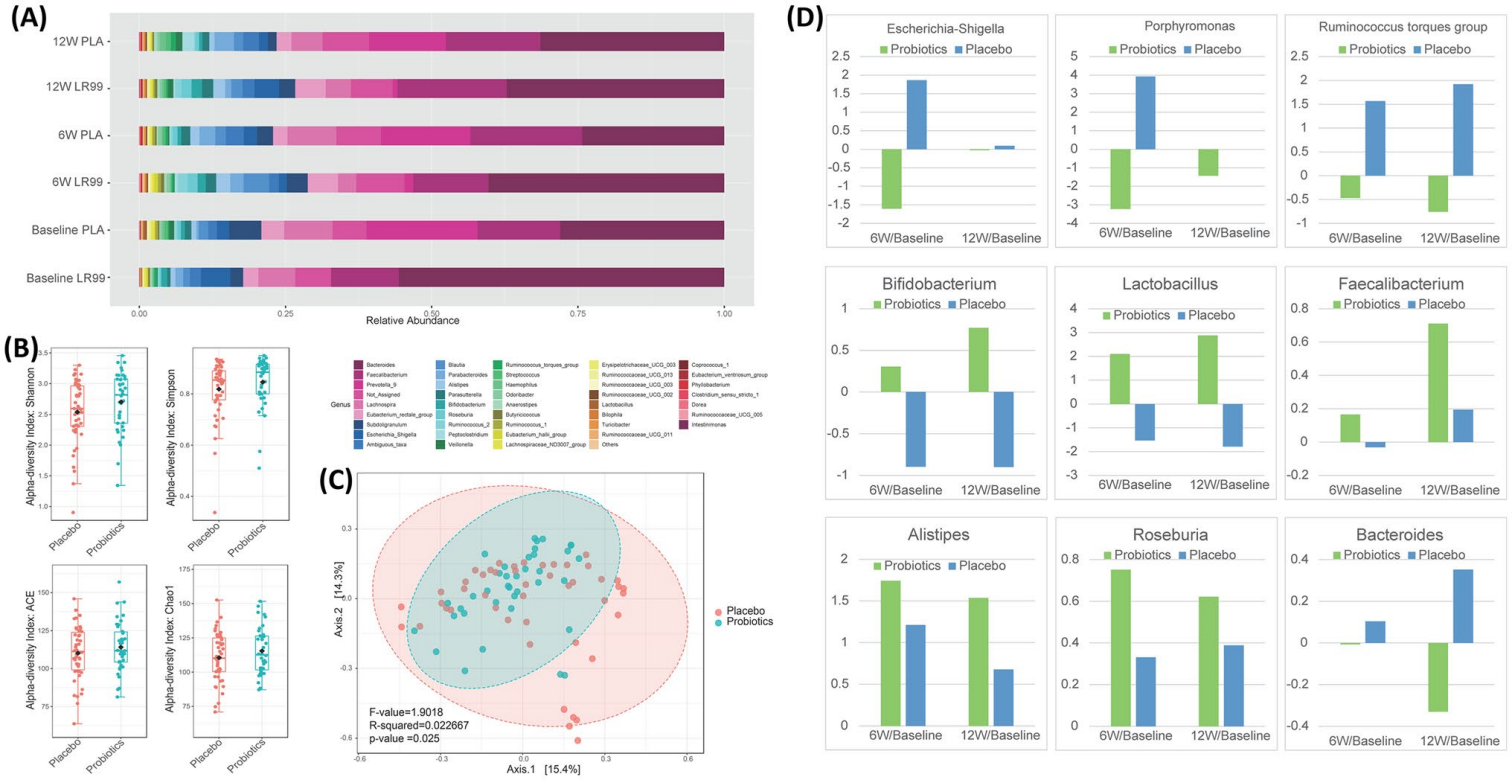
Overview of genus level relative abundances and measures of microbial diversity. **A** Relative abundance plots of the gut microbiota at baseline, 6 weeks, and 12 weeks at the genus level. **B** Mean α-diversity measured via Shannon, Simpson, ACE, and Chao1 indices. **C** β-diversity with principal coordinates analysis (PCoA) score plots of gut microbial data based on a Bray–Curtis dissimilarity matrix. **D** Summary of the top 9 most prominent fold changes of relative abundance at genus level over the course of intervention for the probiotics group (green) and placebo (blue). Each bar represents the log 2-transformed relative change of gut microbial abundance of 6 and 12 weeks compared with the baseline

After sequencing, we obtained a total of 3,198,401 raw reads and an average of 49,206.169 reads per sample (ranging from 29,501 to 71,027 reads per sample). Phylum and genus level variations in gut microbiota composition over the intervention course are shown in Fig. 2A for both the probiotics and placebo groups.

Overall, α-diversity determined using Shannon, Simpson, ACE, and Chao1 indices did not show any significant group differences (Fig. 2B). However, β-diversity showed a significant separation with probiotics treatment via permutational multivariate ANOVA (PERMANOVA, F = 1.9018; *R*^*2*^ = 0.022667; *P* < 0.05, Fig. 2C).

To characterize the change in abundance of potentially clinically significant bacteria over the intervention course, we calculated the log twofold changes of detected and identified gut microbiota. The top 9 most prominent fold changes of gut microbiota abundance agglomerated at the genus level are presented in Fig. 2D.

In an attempt to elucidate the changes in the gut microbiome functional profile between those receiving active probiotic and those receiving placebo, we applied predictive functional profiling and performed groupwise comparisons of mean abundance differences for each identified functional pathway. Several functional pathways were determined to be differentially expressed among subjects receiving active probiotic (Table 4, *Q* < 0.1).

**Table 4:** The predicted KEGG enzyme abundance based on PICSRUSt-2 predictive functional profiling for subjects receiving either active probiotic or placebo control. The average abundance of KEGG enzyme abundances is differentially enriched in placebo and probiotics at level 3

Feature	Mean abundance		Mean Difference (A–C)	Mean ratio (A/C)	*P*-Value	*Q-*Value
	Active probiotic	Placebo control				
Arachidonic acid metabolism	218.95	360.13	−141.19	0.61	< 0.001	0.0038
Valine, leucine, and isoleucine biosynthesis	7469.34	6922.96	546.37	1.08	0.0002	0.0067
Meiosis (yeast)	55.66	27.30	28.36	2.04	0.0013	0.0410
Flavonoid biosynthesis	49.18	20.94	28.23	2.35	< 0.001	0.0038
Carotenoid biosynthesis	71.61	20.88	50.73	3.43	< 0.001	< 0.001
Steroid biosynthesis	14.11	2.17	11.94	6.51	< 0.001	0.0038
Various types of N-glycan biosynthesis	2.94	0.04	2.91	84.02	< 0.001	< 0.001
Photosynthesis (antenna proteins)	90.51	0.47	90.04	192.58	< 0.001	< 0.001
Calcium signaling pathway	5.32	0.03	5.30	209.20	< 0.001	< 0.001

**Fig. 3:**
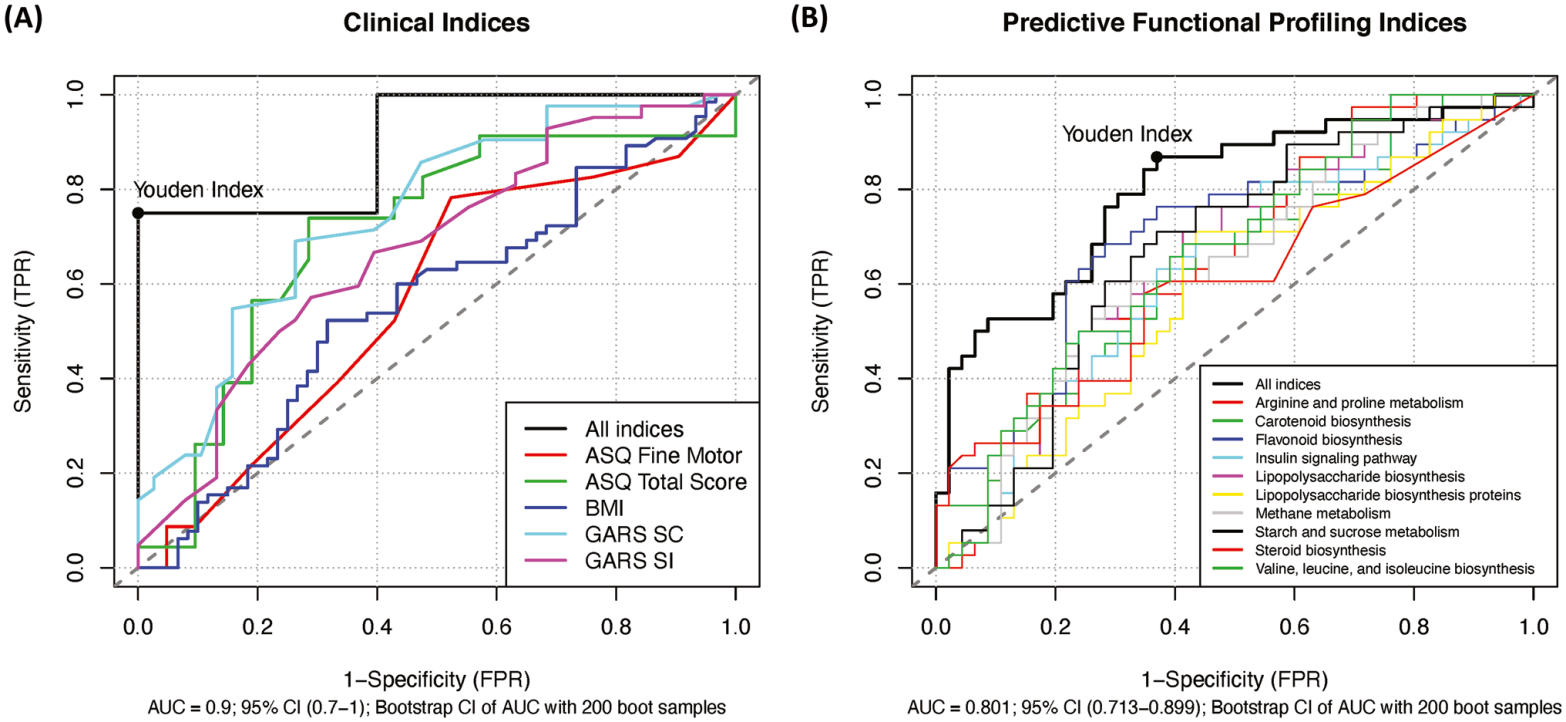
ROC curve of classification between treatment and placebo groups based on select clinical indices and functional metagenomic features using logistic regression. **A** Classification using clinical indices, including ASQ-3 total and fine motor scores and GARS-3 SC and SI scores. **B** Classification using select functional features of the gut metagenome

Subsequently, using receiver operated characteristic (ROC) curve analysis, we identified the significant clinical parameters (BMI, social communication, social interaction, total ASQ, fine motor) that can be used as biomarkers for treatment response and characterize subjects either receiving probiotics or placebo (Fig. 3A); the fitted logistic regression model is summarized as a table in Online Resource 3. We then identified several key metagenomic functional pathways that can be used to characterize subjects receiving either active probiotics or placebo (Fig. 3B); the fitted logistic regression model is summarized as a table in Online Resource 4. Classification using clinical indices, including ASQ-3 total and fine motor scores and GARS-3 SC and SI scores, resulted in an AUC of 0.9 (95% CI = 0.7–1). Similarly, classification using select functional features of the gut metagenome resulted in an AUC of 0.801 (95% CI = 0.713–0.899).

### Correlation Between Gut Microbiota Abundances and Clinical Indices

Associations between family and genus level microbiota abundances and clinical indices were assessed via MaAsLin2 as univariate linear correlations. Significant correlations at the family and genus levels against clinical indices for measurements at weeks 6 and 12 combined are reported in Table 5. Specifically, BMI is negatively associated with Bifidobacteriaceae (*R* = −0.431, *Q* < 0.1) and positively associated with Erysipelotrichaceae family level relative abundances (*R* = 0.261, *Q* < 0.1). Standardized (z-score) of BMI is negatively associated with *Faecalibacterium* (*R* = −0.546, *Q* < 0.1) and positively associated with *Subdoligranulum* genus level relative abundances (*R* = 0.641, *Q* < 0.1). *Alistipes* genus level relative abundance is found to be negatively associated with both GARS-3 ER (*R* = −0.644, *Q* < 0.1) and RRB scores (*R* = −0.595, *Q* < 0.1).

**Table 5:** Summary of significant correlations between genus and family level bacterial abundance and clinical measurements at weeks 6 and 12 combined in the active probiotic group. Taxonomic ranking is labeled in parentheses with “f” denoting family level and “g” denoting genus level microbiota

Microbiota	Clinical feature	Coefficient	Standard error	*P*-Value	*Q*-Value
Bifidobacteriaceae (f)	BMI	−0.431	0.12685	0.00433	0.06707
Erysipelotrichaceae (f)	BMI	0.261	0.07384	0.00329	0.06707
*Alistipes* (g)	ER	−0.644	0.11926	0.00043	0.01217
*Alistipes* (g)	RRB	−0.595	0.14462	0.00262	0.07335
*Faecalibacterium* (g)	BMI (z-score)	−0.546	0.16112	0.00438	0.09727
*Subdoligranulum* (g)	BMI (z-score)	0.641	0.2027	0.00695	0.09727

## Discussion

In our 12-week, randomized, double-blind, placebo-controlled trial of 71 subjects with PWS, *Lact. reuteri* LR-99 significantly reduced the BMI in those receiving the active probiotic compared to those receiving placebo at both 6 weeks (*P* < 0.05) and 12 weeks (*P* < 0.01) of the treatment. One recent study found that *Lact. reuteri* administration in the PWS population improved insulin sensitivity and decreased abdominal adiposity in children older than 4.5 years of age [15]. However, the mechanisms involved in the induction of such effects remain unclear and may manifest in many different bacteria. The present study provides new evidence for *Lact. reuteri* as a potential, early therapeutic option for PWS that may prevent obesity and related complications. Furthermore, we hope that the study can shed light on the effects and associated mechanistic role of LR-99 in individuals with PWS.

PWS individuals have been found to have absolute or functional growth hormone (GH) deficiency, and GH replacement is currently the most effective treatment for PWS [3]. GH was found not only to increase height, but also decrease body fat and improve cognition, motor, and mental functions [3]. With earlier initiation of GH treatment, increased efficacy and prognostic benefit have been observed [3]. One study has found that probiotics *Lact. reuteri* could increase growth hormone level in mice [31], which reveals a potential mechanism by which probiotics can improve growth and reduce BMI in PWS patients via promotion of endogenous growth hormone release. Our findings warrant further investigation into the biological mechanisms of probiotics, a promising intervention for PWS with better tolerance and convenience than GH replacement.

Interestingly, we found that *Lact. reuteri* intervention significantly improved social communication (*P* < 0.01) and social interaction (*P* < 0.05) compared to controls for those older than 3 years old. Moreover, we found a significant increase in total ASQ-3 score (*P* < 0.05) and fine motor sub-scale (*P* < 0.05) in *Lact. reuteri* intervention group compared with placebo control group when compared at the last study visit (week 12). While a significant groupwise difference is observed for social communication at baseline (week 0, *P* < 0.05), such differences are observed with a greater magnitude between groups at week 12 (*P* < 0.01), which is likely a result of *Lact. reuteri* supplementation. As previously mentioned, *Lact. reuteri* has been reported to upregulate oxytocin in mice and increase the abundance of OXT-producing cells in the caudal PVN of the hypothalamus in humans [17, 19, 20]. Through vagus nerve-mediated modulation of oxytocin signaling, *Lact. reuteri* was found to rescue social deficits in multiple mouse models of ASD [20]. Effective treatment of PWS subjects using oxytocin nasal spray in the past [32] and recently in an unpublished phase III CARE-PWS trial provides additional evidence that the improvements we observed in social communication may be related to *Lact. reuteri*-induced endogenous oxytocin upregulation [32, 33]. These findings of social improvement by *Lact. reuteri* have not been reported in humans previously, which warrants further study of potential oxytocin signaling deficits involved in the pathogenesis of PWS and other developmental disorders that may also be improved through probiotic supplementation.

The microbiome composition changes we observed with the intervention have been previously linked to weight reduction and inflammatory attenuation. Notably, we found a significant separation of the gut microbiome β-diversity between the probiotics and the placebo group after treatment. β-Diversity has been directly correlated with long-term weight loss when adhering to a controlled diet [34]. Significant alteration of microbiome composition in PWS by probiotics has not been reported previously. In fact, administration of the probiotic *Bifidobacterium animalis* spp. *lactis* in the PWS population was found to have no significant effect on microbiome composition [11]. Therefore, more research about the variable effects of different probiotic strains on obesity is warranted.

After administration of *Lact. reuteri*, we also noted a trend of reduction in the abundance of several bacteria including *Escherichia-Shigella*, *Porphyromonas*, *Ruminococcus torques*, and *Bacteroides. Escherichia-Shigella* is well recognized pathogenic bacteria and is found to be enriched in individuals with obesity and type 2 diabetes [35], in addition to those with autism and co-morbid constipation [36]. The role of periodontal pathogens, including *Porphyromonas gingivalis* (*P. gingivalis*), in the onset or exacerbation of systemic diseases, has been demonstrated [37]. *Ruminococcus torques* is one of the prominent species enriched in people with irritable bowel disease [38]. *Bacteroides* was found to be enriched in subjects with type 1 diabetes [39]. However, the role of *Bacteroides* in the pathology of type 2 diabetes and inflammation remains controversial [40, 41].

Conversely, *Bifidobacterium*, *Lactobacillus*, *Faecalibacterium*, *Roseburia*, and *Alistipes* each trended towards increased abundance in the gut after *Lact. reuteri* treatment. *Lactobacillus*, the genus to which the interventional probiotic belongs, has protective effects against weight gain in humans [42]. *Bifidobacterium* is widely regarded as beneficial to gut health and weight reduction [43, 44]. *Alistipes*, although its abundance has been inversely correlated to adiposity, lipid, and glucose homeostasis parameters, [45] has been both negatively and positively associated with to autism spectrum disorders and remains controversial [46, 47]. *Roseburia* and *Faecalibacterium* are butyrate-producing, anti-inflammatory bacteria. *Roseburia* was reported to affect colonic motility, immunity maintenance, and anti-inflammatory properties in various metabolic pathways and several diseases, including irritable bowel syndrome, obesity, type-2 diabetes, nervous system conditions, and allergies [48]. *Faecalibacterium* was found to decrease gut permeability and inflammation [49].

Our findings about the differential abundance of bacterial genera following probiotic treatment interestingly overlapped with the findings from a recent study about the microbiome composition of people with PWS [6]. While they found that *Bacteroides* was enriched in overweight PWS patients relative to normal weight controls, our study found that *Bacteroides* was decreased after probiotics treatment. However, their study did find that *Escherichia* was reduced and *Bifidobacterium* was enriched in overweight PWS patients, while our study found that *Lact. reuteri* further reduced and increased the abundance of these probiotics, respectively. These results indicate that different bacterial genera may have a variable contribution to PWS pathogenesis and warrants further investigation.

Furthermore, by using predictive functional gene analysis, we found significant upregulation of calcium signaling, flavonoid biosynthesis, carotenoid biosynthesis, steroid biosynthesis, N-glycan biosynthesis, valine, leucine, and isoleucine biosynthesis with both *P-* and *Q*-values < 0.05. Of note, carotenoids, a type of antioxidant, were previously found to have beneficial effects on obesity and obesity-associated pathologies [50]. Further, dietary supplementation with Leu or Ile reduced body weight by regulating lipid metabolism-related genes and insulin sensitivity and alleviated hepatic steatosis [51].

The insulin signaling pathway and starch and sucrose metabolism were also found to be upregulated with *P* < 0.05, but *Q* > 0.1. This non-significant trend provides additional evidence in support of the findings by Amat-Bou et al. that *Lact. reuteri* improves insulin signaling and, in this way, promotes normal weight [15]. However, while the probiotic study conducted by Amat-Bou et al. demonstrated such positive effects in individuals with PWS using the *Bifidobacterium* strain BPL1, it is unsurprising that such effects are also observed in the present study of LR-99, as both Bifidobacteria and Lactobacilli have important interconnected functional roles in the gut such as fructose and oligosaccharide metabolism [52]. Further, Peng et al. found that carbohydrate intake, unlike dietary fat or protein, was correlated to changes in microbiome diversity and composition in PWS patients [6]. These results indicate that carbohydrate consumption and metabolism may be keys to the pathogenesis of PWS and the efficacy of probiotic treatment.

The predictive functional gene analysis also showed the significant downregulation of arachidonic acid metabolism with both *P* and *Q* < 0.05. Lipopolysaccharide (LPS) and phosphotransferase system (PTS) were also found to be downregulated with *P* < 0.05 but *Q* > 0.05. Lipopolysaccharide (LPS), endotoxin from gram-negative pathogenic bacteria such as *Escherichia-Shigella*, has been reportedly involved in the development of obesity and autism.[53, 54] Taken together, the microbiome composition data and predictive functional gene analysis indicate that the diversity separation caused by *Lact. reuteri* probiotics treatment favors protection against inflammation, obesity, metabolic syndrome, and ASD.

Using ROC curve analysis, we found the clinical indices, including ASQ-3 total, fine motor scores, GARS-3 SC, and SI scores, resulted in an AUC of 0.9 (95% CI = 0.7–1). Classification using select functional features of the gut metagenome resulted in an AUC of 0.801 (95% CI = 0.713–0.899). The high sensitivity and specificity by which improved clinical indices and changes in gut microbiome composition can distinguish PWS who received *Lact. reuteri* serves as strong evidence to the efficacy of this probiotic treatment. RRB is one of the core symptoms of ASD and has been reported in as many as 25–40% of PWS cases [55]. *Alistipes* was found to be negatively correlated with RRB; a decrease in the relative abundance of *Alistipes* was found in ASD which is consistent with our finding [46, 54]. *Subdoligranulum* was found to be positively correlated with BMI, while *Faecalibacterium* was negatively correlated with BMI. *Subdoligranulum* was found to be increased in obese mice [56], while individuals with obesity have decreased abundance of *Faecalibacterium* [57]. Additionally, *Bifidobacterium* was found to be negatively correlated with BMI, which is expected given its widely recognized effects on promoting gut health and weight reduction [43, 44].

In conclusion, this randomized double-blinded placebo control trial for PWS children showed that treatment with probiotic *Lact. reuteri* for 12 weeks significantly decreased BMI at week 6 and has more pronounced effects when examined after 12 weeks of administration. *Lactobacillus reuteri* administration also significantly improved social communication and interaction, fine motor function, and overall development score at week 12 in young children. These novel findings have vital implications for early treatment in PWS. Probiotic treatment also altered microbiome composition and function to favor anti-obesity, anti-inflammation, and influence brain function. The significantly improved clinical indices and functional features of the gut metagenome as a result of probiotic treatment were each found to have predictive value with high specificity and sensitivity.

There are some limitations to the study that deserve consideration. First, despite our adoption of proper recruitment and retention strategies, PWS participant enrolment and retention for this trial were challenging; the sample size was relatively small and limited further subgroup analysis. Second, the broad age range used in this study resulted in high subject population heterogeneity and potentially variable treatment efficacy. Third, assessment of fecal microbiome was not controlled for dietary habits and other relevant environmental factors, which may influence the microbial abundances at the individual level. Thus, future studies with larger sample sizes, improved control for environmental factors, and subgroup stratification are warranted. Due to the limitations of the study listed above, further studies are warranted to investigate the mechanism and efficacy of LR-99 probiotic treatment in PWS.

## Supplementary Information

Below is the link to the electronic supplementary material.

Supplementary file1 (PDF 226 KB)

Supplementary file2 (PDF 92 KB)

Supplementary file3 (PDF 90 KB)

Supplementary file4 (PDF 93 KB)

## Data Availability

All 16 s rRNA Illumina amplicon sequencing data presented in this study are openly available in The National Centre for Biotechnology Information (NCBI) Sequence Read Archive (SRA) with accession number PRJNA643297.

## Code Availability

Available upon request.
