# Supplementary material for: The Effects of Limosilactobacillus reuteri LR-99 Supplementation on Body Mass Index, Social Communication, Fine Motor Function, and Gut Microbiome Composition in Individuals with Prader–Willi Syndrome: a Randomized Double-Blinded Placebo-Controlled Trial
Source: Probiotics Antimicrob Proteins. 2021 Jun 11;13(6):1508–20. doi: 10.1007/s12602-021-09800-9 (PMC8578098; doi:10.1007/s12602-021-09800-9)
Supplement: Supplementary file 1 — Supplementary file1 (PDF 226 KB) [file 12602_2021_9800_MOESM1_ESM.pdf]

The effects of *Limosilactobacillus reuteri* LR-99 supplementation on body mass index, social communication, fine motor function, and gut microbiome composition in individuals with Prader-Willi Syndrome: a randomized double-blinded placebo-controlled trial

Xue-Jun Kong [1,2,*], Kevin Liu [1], Patrick Zhuang [1], Ruiyi Tian [1], Siyu Liu [1], Cullen Clairmont [1], Xiaojing Lin [3], Hannah Sherman [1], Junli Zhu [4], Yelan Wang [1], Michelle Fong [1], Alice Li [1], Bryan K. Wang [5], Jinghan Wang [6], Zhehao Yu [7], Chen Shen [7], Xianghua Cui [7], Hanyu Cao [7], Ting Du [7], Guobin Wan [8] and Xia Cao [7]

1 Athinoula A. Martinos Center for Biomedical Imaging, Massachusetts General Hospital, Boston, MA
2 Department of Medicine and Psychiatry, Beth Israel Deaconess Medical Center, Boston, MA
3 PWS Care and Support Center, Hangzhou, China
4 Yale University, New Haven, CT, USA
5 Brandeis University, Waltham, MA, USA
6 New York University, New York, NY, USA
7 The Second Affiliated Hospital of Kunming Medical University, Kunming, Yunnan, China
8 Shenzhen Maternity and Child Healthcare Hospital, Shenzhen, Guangdong, China
* Correspondence: xkong1@mgh.harvard.edu

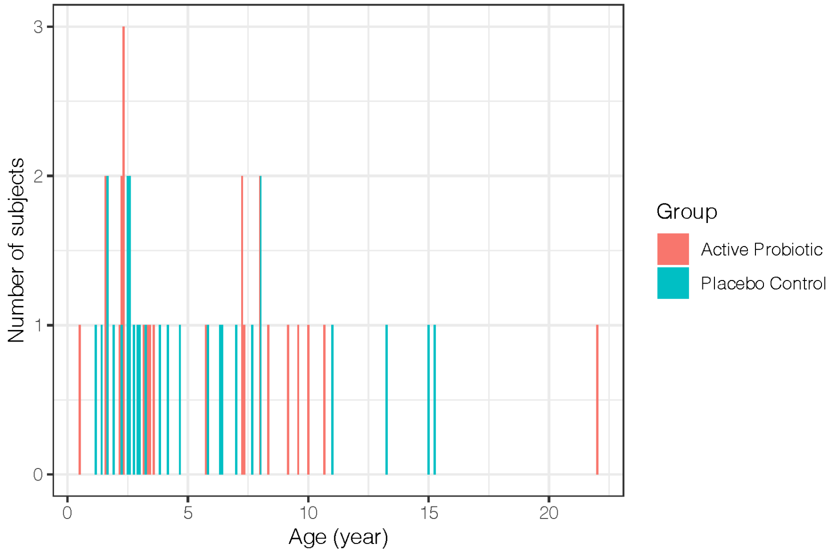

Online Resource 1. Study participant age distribution. Participant groups are indicated by the color of the frequency bar. The age of subjects receiving placebo control ranges from 1 to 15 years while those receiving the active probiotic have ages ranging from 0.5 to 23 years.
