# Supplementary material for: The Effects of Limosilactobacillus reuteri LR-99 Supplementation on Body Mass Index, Social Communication, Fine Motor Function, and Gut Microbiome Composition in Individuals with Prader–Willi Syndrome: a Randomized Double-Blinded Placebo-Controlled Trial
Source: Probiotics Antimicrob Proteins. 2021 Jun 11;13(6):1508–20. doi: 10.1007/s12602-021-09800-9 (PMC8578098; doi:10.1007/s12602-021-09800-9)
Supplement: Supplementary file 2 — Supplementary file2 (PDF 92 KB) [file 12602_2021_9800_MOESM2_ESM.pdf]

The effects of *Limosilactobacillus reuteri* LR-99 supplementation on body mass index, social communication, fine motor function, and gut microbiome composition in individuals with Prader-Willi Syndrome: a randomized double-blinded placebo-controlled trial

Xue-Jun Kong [1,2,*], Kevin Liu [1], Patrick Zhuang [1], Ruiyi Tian [1], Siyu Liu [1], Cullen Clairmont [1], Xiaojing Lin [3], Hannah Sherman [1], Junli Zhu [4], Yelan Wang [1], Michelle Fong [1], Alice Li [1], Bryan K. Wang [5], Jinghan Wang [6], Zhehao Yu [7], Chen Shen [7], Xianghua Cui [7], Hanyu Cao [7], Ting Du [7], Guobin Wan [8] and Xia Cao [7]

1 Athinoula A. Martinos Center for Biomedical Imaging, Massachusetts General Hospital, Boston, MA
2 Department of Medicine and Psychiatry, Beth Israel Deaconess Medical Center, Boston, MA
3 PWS Care and Support Center, Hangzhou, China
4 Yale University, New Haven, CT, USA
5 Brandeis University, Waltham, MA, USA
6 New York University, New York, NY, USA
7 The Second Affiliated Hospital of Kunming Medical University, Kunming, Yunnan, China
8 Shenzhen Maternity and Child Healthcare Hospital, Shenzhen, Guangdong, China
* Correspondence: xkong1@mgh.harvard.edu

Online Resource 2. Estimated marginal means of BMI at each study timepoint.

Intervention	Study Visit	Estimated Means (95% CI)	Standard Error	Degrees of Freedom
Active Probiotic	0-weeks	20.28 (18.347; 22.212)	0.732	18.074
	6-weeks	19.300 (17.34; 21.261)	0.754	21.041
	12-weeks	18.988 (17.049; 20.928)	0.743	20.146
Placebo Control	0-weeks	19.455 (15.751; 23.159)	1.427	21.507
	6-weeks	21.908 (17.249; 26.567)	1.838	30.166
	12-weeks	17.996 (13.252; 22.739)	1.873	30.510
