# Supplementary material for: The Effects of Limosilactobacillus reuteri LR-99 Supplementation on Body Mass Index, Social Communication, Fine Motor Function, and Gut Microbiome Composition in Individuals with Prader–Willi Syndrome: a Randomized Double-Blinded Placebo-Controlled Trial
Source: Probiotics Antimicrob Proteins. 2021 Jun 11;13(6):1508–20. doi: 10.1007/s12602-021-09800-9 (PMC8578098; doi:10.1007/s12602-021-09800-9)
Supplement: Supplementary file 3 — Supplementary file3 (PDF 90 KB) [file 12602_2021_9800_MOESM3_ESM.pdf]

The effects of *Limosilactobacillus reuteri* LR-99 supplementation on body mass index, social communication, fine motor function, and gut microbiome composition in individuals with Prader-Willi Syndrome: a randomized double-blinded placebo-controlled trial

Xue-Jun Kong [1,2,*], Kevin Liu [1], Patrick Zhuang [1], Ruiyi Tian [1], Siyu Liu [1], Cullen Clairmont [1], Xiaojing Lin [3], Hannah Sherman [1], Junli Zhu [4], Yelan Wang [1], Michelle Fong [1], Alice Li [1], Bryan K. Wang [5], Jinghan Wang [6], Zhehao Yu [7], Chen Shen [7], Xianghua Cui [7], Hanyu Cao [7], Ting Du [7], Guobin Wan [8] and Xia Cao [7]

[1] Athinoula A. Martinos Center for Biomedical Imaging, Massachusetts General Hospital, Boston, MA
[2] Department of Medicine and Psychiatry, Beth Israel Deaconess Medical Center, Boston, MA
[3] PWS Care and Support Center, Hangzhou, China
[4] Yale University, New Haven, CT, USA
[5] Brandeis University, Waltham, MA, USA
[6] New York University, New York, NY, USA
[7] The Second Affiliated Hospital of Kunming Medical University, Kunming, Yunnan, China
[8] Shenzhen Maternity and Child Healthcare Hospital, Shenzhen, Guangdong, China
* Correspondence: xkong1@mgh.harvard.edu

Online Resource 3. Summary of clinical logistic regression model indices used in ROC analysis.

Clinical Index	Estimate	Standard Error	*z*-value	*P*-value
BMI	2.699	2.255	1.197	0.231
GARS-3 Social Communication	0.0271	0.359	0.076	0.940
GARS-3 Social Interaction	0.184	0.217	0.847	0.397
ASQ-3 Total Score	0.102	0.0864	1.184	0.236
ASQ-3 Fine Motor	-0.307	0.263	-1.166	0.244
