# Supplementary material for: The Effects of Limosilactobacillus reuteri LR-99 Supplementation on Body Mass Index, Social Communication, Fine Motor Function, and Gut Microbiome Composition in Individuals with Prader–Willi Syndrome: a Randomized Double-Blinded Placebo-Controlled Trial
Source: Probiotics Antimicrob Proteins. 2021 Jun 11;13(6):1508–20. doi: 10.1007/s12602-021-09800-9 (PMC8578098; doi:10.1007/s12602-021-09800-9)
Supplement: Supplementary file 4 — Supplementary file4 (PDF 93 KB) [file 12602_2021_9800_MOESM4_ESM.pdf]

The effects of *Limosilactobacillus reuteri* LR-99 supplementation on body mass index, social communication, fine motor function, and gut microbiome composition in individuals with Prader-Willi Syndrome: a randomized double-blinded placebo-controlled trial

Xue-Jun Kong [1,2,*], Kevin Liu [1], Patrick Zhuang [1], Ruiyi Tian [1], Siyu Liu [1], Cullen Clairmont [1], Xiaojing Lin [3], Hannah Sherman [1], Junli Zhu [4], Yelan Wang [1], Michelle Fong [1], Alice Li [1], Bryan K. Wang [5], Jinghan Wang [6], Zhehao Yu [7], Chen Shen [7], Xianghua Cui [7], Hanyu Cao [7], Ting Du [7], Guobin Wan [8] and Xia Cao [7]

[1] Athinoula A. Martinos Center for Biomedical Imaging, Massachusetts General Hospital, Boston, MA
[2] Department of Medicine and Psychiatry, Beth Israel Deaconess Medical Center, Boston, MA
[3] PWS Care and Support Center, Hangzhou, China
[4] Yale University, New Haven, CT, USA
[5] Brandeis University, Waltham, MA, USA
[6] New York University, New York, NY, USA
[7] The Second Affiliated Hospital of Kunming Medical University, Kunming, Yunnan, China
[8] Shenzhen Maternity and Child Healthcare Hospital, Shenzhen, Guangdong, China
* Correspondence: xkong1@mgh.harvard.edu

Online Resource 4. Summary of predictive metagenomic profiling logistic regression model indices used in ROC analysis.

Metabolic Pathway	Estimate	Standard Error	*z*-value	*P*-value
Arginine and proline metabolism	1.173E-05	4.019E-05	0.292	0.770
Carotenoid biosynthesis	-7.380E-04	1.362E-03	-0.542	0.588
Flavonoid biosynthesis	-1.331E-04	9.938E-04	-0.134	0.893
Insulin signaling pathway	-7.032E-05	2.345E-04	-0.300	0.764
Lipopolysaccharide biosynthesis	4.598E-05	9.611E-05	0.478	0.632
Lipopolysaccharide biosynthesis proteins	-8.925E-06	6.780E-05	-0.132	0.895
Methane metabolism	7.245E-05	4.201E-05	1.725	0.085
Starch and sucrose metabolism	-4.026E-05	1.812E-05	-2.222	0.026
Steroid biosynthesis	-4.805E-03	3.321E-03	-1.447	0.148
Valine, leucine, and isoleucine biosynthesis	-8.859E-05	5.710E-05	-1.551	0.121
